# Natural Intrauterine Infection with Schmallenberg Virus in Malformed Newborn Calves

**DOI:** 10.3201/eid2008.121890

**Published:** 2014-08

**Authors:** Calixte Bayrou, Mutien-Marie Garigliany, Michael Sarlet, Arnaud Sartelet, Dominique Cassart, Daniel Desmecht

**Affiliations:** University of Liège, Liège, Belgium

**Keywords:** Schmallenberg virus, bovine, calves, malformations, viruses, viral RNA

## Abstract

We surveyed morphologic alterations in calves in Belgium that were naturally infected in utero by Schmallenberg virus (SBV) and born with deformities during January–March 2012. SBV-specific RNA was distributed unevenly in different tissues. Natural intrauterine SBV infection of calves might cause serious damage to the central nervous system and muscles.

During summer and fall 2011, a nonspecific febrile syndrome characterized by hyperthermia and decreased milk production was reported in adult dairy cows from farms in the Netherlands and Germany (*1*,*2*). In November 2011, an enzootic outbreak of abortions, stillbirths, and term births of lambs, kids, and calves that exhibited neurologic signs and/or musculoskeletal malformations emerged throughout northwestern Europe (*3*,*4*). Both syndromes were associated with the presence in the blood (adults) or in the central nervous system (CNS) (newborns) of the genome of a new orthobunyavirus, which was named Schmallenberg virus (SBV) after the place where the first positive samples were collected (*3*,*4*). SBV belongs to the Simbu serogroup (*5*) and, like its phylogenetic relatives Akabane and Aino viruses, can cross the placenta (*6*). Because this new viral disease of ruminants emerged 3 years ago, information is limited. We comprehensively surveyed morphologic alterations in calves naturally infected in utero. In addition, we report the distribution of SBV-specific RNA in the different tissues of these calves, which has implications for diagnosis.

## The Study

In Belgium each year during January–June, field veterinarians refer ≈30 newborn calves per month for necropsy to the University of Liège Faculty of Veterinary Medicine (Liège, Belgium). During January–March 2012, the consequences of SBV infection on bovine fetuses were not yet known, which prompted the staff to look systematically for the new virus in all deformed calves and in calves that died spontaneously without obvious cause. Among the 67 animals in these categories, SBV genetic material was detected in 15 calves by reverse transcription quantitative PCR, and IgG specific for SBV nucleoprotein was systematically highlighted in their serum by ELISA. In addition, all attempts to retrieve the genetic material of bluetongue virus 8 and bovine viral diarrhea virus from the tissues of these 15 seropositive calves failed. None of these calves carried the mutation responsible for noninfectious arthrogryposis in local livestock. These 15 calves, in which both SBV RNA and antibodies against SBV were detected, are the subject of this study.

Detailed information about the methods used to examine the calves is available in Technical Appendix 1. A detailed description of the lesions found in SBV-infected calves is provided in Technical Appendix 2.

**Figure 1:**
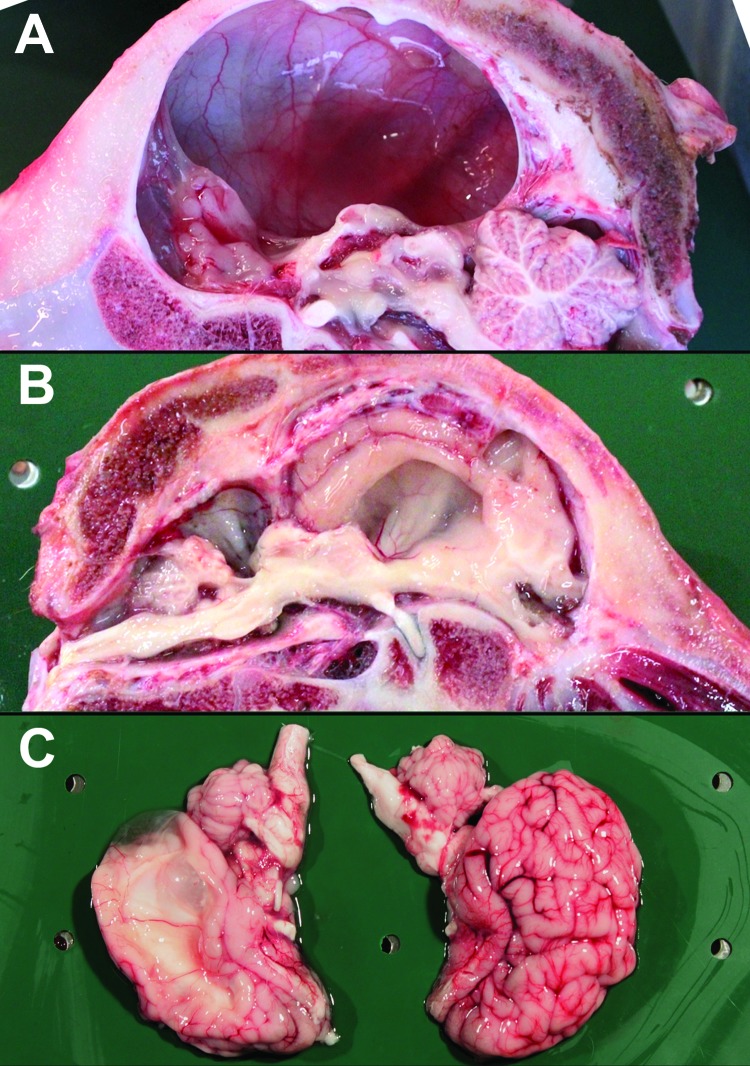
Deformities of the brain in calves naturally infected in utero with Schmallenberg virus, Belgium, January–March 2012. A) Hydranencephaly. B) Hydrocephaly and cerebellar hypoplasia. C) Porencephaly.

SBV-positive animals weighed significantly less than expected (32 kg + 15 kg vs. 49 kg + 4 kg, p<0.05). The body mass deficit, severity of deformities in whole-body conformation, and amount of skeletal muscle were obviously correlated (Table 1; Technical Appendix 3 Figure 1.

**Table 1:** Macroscopic characteristics of 15 SBV-infected newborn calves at necropsy, Belgium, January–March 2012*

Characteristic	WBD-0†	WBD-1†	WBD-2†	WBD-3†	Total no. calves
No. calves	2	4	4	5	15
Method of death					
Euthanasia	2	3	0	0	5
Spontaneous	0	1	4	5	10
Bodyweight, kg‡§	49 ± 4	39 ± 3	34 ± 3	21 ± 2	
Axial musculoskeletal system					
Defect location					
Cervical	0	2	4	5	11
Thoracic	0	0	2	5	7
Lumbar	0	0	0	5	5
Type of deviation					
Lateral	0	2	4	5	11
Dorso-ventral	0	0	1	4	5
Helicoidal	0	0	1	4	5
Appendicular musculo-skeletal system					
Arthrogryposis (>1 limb involved)	0	3	4	5	12
Symetric limb involvement	NA	3	3	5	11
Forelimb/hind limb involvment	NA	0	1	5	6
Forelimbs only	NA	2	3	0	5
Hind limbs only	NA	1	0	0	1
Head					
Coaptation defect					
Prognathism	0	0	0	1	1
Brachygnathism	0	1	1	2	4
Altered profile					
Horse-like	0	1	1	0	2
Pig-like	0	0	0	2	2
Broken sagittal axis	0	1	2	2	5
Central nervous system					
Porencephaly	2	3	3	1	9
Hydranencephaly	1	1	0	1	3
Hydrocephaly	0	0	1	4	5
Cerebellar hypoplasia	0	0	0	1	1
Micromyelia	2	4	4	5	15

*Fifteen newborn calves infected in utero by SBV were categorized according to the extent of their deformities. The table lists the gross morphologic changes observed at necropsy. SBV, Schmallenberg virus; WBD, whole-body deformity score; NA, not applicable. †Animals with neurologic signs and apparently normal body shape were given a WBD of 0; those with altered body shape were scored 1, 2, or 3 depending on whether 1, 2, or 3 skeletal segments were deformed, respectively (spine, forelimbs, or hind limbs). All values are number of calves unless otherwise indicated. ‡Mean + SD. §Bodyweight of age-matched Belgian Blue healthy calves is 49.2 kg + 7.1 kg (*7*).

**Figure 2:**
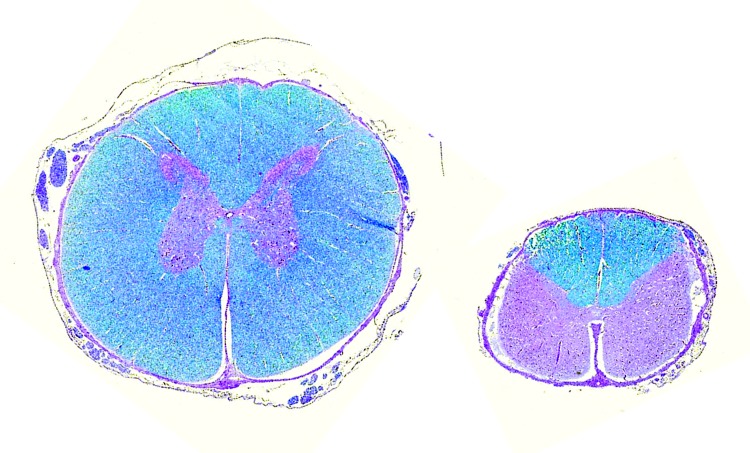
Micromyelia. Age- and site-matched spinal cord transversal histologic section at the level of C4. Left, control calf; right, Schmallenberg virus (SBV)–infected calf. Note atrophy/hypoplasia and prominent deficiency of stainable myelin in ventral and lateral tracts of SBV-infected calf. Luxol fast blue staining.

We observed overall permanent deviations of the axial skeleton in all 3 planes (Technical Appendix 3 Figure 2), the most common being a lateral deviation of the cervical spine (torticollis). In the most distorted animals, the torticollis was accompanied by a dorso-ventral deviation of the thoracolumbar spine. Most SBV-infected calves displayed joint fixation of 1 or all joints of >1 limbs (arthrogryposis). Tendons spanning fixed joints were shorter than expected, and corresponding muscles displayed decreased mass and altered color. Often the animal’s head was distorted, having a horse-like or pig-like shape, brachygnathism, prognathism, and/or diverging sagittal axes (online Technical Appendix 3 Figure 3).

We systematically observed major alterations after we opened the skull and spinal canal (Figure 1; Technical Appendix 3 Figure 4). These changes involved the spinal cord and the telencephalon, whereas the brainstem and cerebellum were kept intact (although 1 cerebellum was hypoplastic). We consistently observed a decrease in the cross-sectional area of the spinal cord (Figure 2), which correlated positively with the magnitude of axial/appendicular musculoskeletal deformities (Technical Appendix 3 Figure 5). The neopallial part of the telencephalon was always decreased, giving 3 distinct morphotypes. In some calves, we detected multiple, bilateral, and randomly located cystic cavities, most of which communicated with the ipsilateral ventricle (porencephaly). In other cases, all that remained from the neopallium was a thin, nearly transparent membrane, sometimes with a few floating smooth-surfaced islets resembling cortex (hydranencephaly). Finally, in a third subset of calves, the brain appeared normal, but we observed a severe, bilateral, and symmetric dilatation of lateral ventricles after section (hydrocephaly).

Microscopic examination of the spinal cord revealed a significant decrease in neuron numbers, the magnitude of which correlated positively with the severity of whole-body deformities (Table 2). Muscle sections displayed a diffuse pattern of increased fiber size variation with connective tissue and adipocyte infiltrations (Technical Appendix 4 Table 1).

**Table 2:** Correlation between spinal neuron counts and axial muscle histologic changes in 15 SBV-infected newborn calves, Belgium, January–March 2012*

Structure examined	Control calves	WBD/calf ID																	
		WBD-0			WBD-1					WBD-2					WBD-3				
		A	B		C	D	E	F		G	H	I	J		K	L	M	N	O
Axial muscles, histology																			
Musculus semispinalis capitis, cas	0	NT	0		0	0	3	0		3	3	NT	3		3	3	3	3	3
Musculus semispinalis capitis, ces	0	NT	1		0	0	2	0		3	3	NT	3		3	3	3	3	3
Spinal cord																			
Dorsal horn neurons, no.																			
Left dorsal horn	12 + 5	NT	11		21	4	5	10		5	1	NT	4		0	0	0	7	3
Right dorsal horn	11 + 4	NT	23		17	6	5	10		7	3	NT	5		1	0	0	5	0
Ventral horn neurons, no.																			
Left ventral horn.	50 + 10	NT	56		57	15	1	45		9	1	NT	0		0	0	0	7	0
Right ventral horn	50 + 4	NT	47		55	14	3	31		7	0	NT	0		0	0	0	4	0

*Extent of histologic changes was reported semiquantitatively by using a score of 0, 1, 2, or 3 depending on whether the histologically normal tissue extended over 100%, 75%–100%, 25%–75%, or <25% of the area examined and intensity of shading reflects these values. Neurons were enumerated in transverse sections of the spinal cord corresponding to C4. Intensity of shading reflects the magnitude of neuron deficits. SBV, Schmallenberg virus; WBD, whole-body deformity score; NT, not tested; cas, concave side; ces, convex side.

The viral RNA was always present in the CNS and sometimes in the lungs and colon (Technical Appendix 4 Table 2). When the entire cohort was considered, SBV was detected in all parts of the CNS. When we examined the animals individually, however, the detection rate varied depending on the segment (Technical Appendix 4 Table 2). The virus was almost always detected in the spinal cord (93%) and the neopallium (87%); often in the midbrain (83%) and pons (67%); and about half the time in the diencephalon, cerebellum, and paleopallium. The practical implications of these findings for routine diagnosis are highlighted separately (Technical Appendix 5).

## Conclusions

Our findings show that natural in utero infection of the bovine fetus by SBV may result in serious damage to the CNS and muscles. Mechanistic hypotheses that could explain these alterations are discussed in Technical Appendix 6. Similar to the situation with Akabane virus infection (*8*), the clinical picture shown by in utero SBV-infected newborn calves is likely to depend largely on the age of the fetus at the time of infection. The infection must be quickly contained if the fetus is infected when immunocompetent (>120–150 days after conception), and we deduce that damages inflicted by the virus consequently have no or little effect on its further development. Conversely, the infection probably spreads more easily and lasts much longer if the virus contaminates an immunologically immature fetus. Because transplacental infection is possible only when the first placentome is present (30 days after conception in cattle), the window during which the infection of a bovine fetus might lead to a porencephaly/hydranencephaly-micromyelia-arthrogryposis syndrome ranges from 30 to 150 days after conception. The degree of overall body deformity correlated with a progressively greater reduction in the size of the spinal cord (as determined by spinal cord:foramen magnum ratio) and with fewer spinal neurons, suggesting that the lack of movement leading to arthrogryposis results directly from the spinal cord lesions, leading to denervation atrophy of skeletal muscle. This primary role for the spinal cord lesion is further supported by the tendency of forelimbs and hind limbs to be affected bilaterally because muscle involvement might be expected to lead to more randomly distributed lesions.

When SBV virus infects the bovine fetus during the risk window mentioned above and causes neuromuscular defects, its genetic material remains detectable at term—thus 4 months later—at a minimum. The physical form of this persisting virus and the way it persists in the face of the seroconversion are unknown. The hypothesis of the existence of sites of persistence must be addressed, for example, in the CNS (≈90% of cases were virus positive at term) or lungs (≈30%). In practice, a priority is to establish whether SBV persists in calves infected in utero but born asymptomatic.

Technical Appendix 1 Materials and methods.

Technical Appendix 2 Gross pathology and histology.

Technical Appendix 3 Definition of whole-body deformity (WBD) scores in Schmallenberg virus (SBV)–infected newborn calves, Belgium, 2012; deformities of the axial skeleton ; deformities of the head ; micromyelia ; occipital ratios (mean ± SD) in control and SBV-positive calves ; and end-stage muscles in a typical SBV-infected calf.

Technical Appendix 4 Distribution of microscopic lesions and virus-specific RNA in the skeletal mucles of 15 Schmallenberg virus–infected newborn calves ; distribution of microscopic lesions and virus-specific RNA in the central nervous system ; and distribution of microscopic lesions and virus-specific RNA in thoraco-abdominal organs.

Technical Appendix 5 Diagnostic considerations.

Technical Appendix 6 Mechanistic hypotheses underlying central nervous system and muscle changes.
