# Supplementary material for: Natural Intrauterine Infection with Schmallenberg Virus in Malformed Newborn Calves
Source: Emerg Infect Dis. 2014 Aug;20(8):1327–30. doi: 10.3201/eid2008.121890 (PMC4111192; doi:10.3201/eid2008.121890)
Supplement: Technical Appendix 1 — Materials and methods. [file 12-1890-Techapp-s1.pdf]

Article DOI: http://dx.doi.org/10.3201/eid2008.121890

Natural Intrauterine Infection with Schmallenberg Virus in Malformed Newborn Calves

Technical Appendix 1

Materials and Methods

Context

Field veterinarians refer ≈2 200 dead animals per year for necropsy to the University of Liège Faculty of Veterinary Medicine. For cattle, a dedicated shuttle runs daily throughout Wallonia (Belgium) to remove and transport the bodies from the farms to the Faculty, which enables necropsy within a reasonable time, 12–48 hours after death in 90% of cases. During an ≈6-month period beginning in January 2012, an unusual musculoskeletal syndrome event was recognized among referred newborn calves. The affected animals were born at term, without dystocia, and none of them had suckled or received colostrum. They displayed mild to severe deformities of axial and appendicular systems, and corresponding anamnestic data consistently mentioned severe antemortem behavioral and motor dysfunctions. The frequency of such cases was ≈20 times that expected based on the 1980–2010 period.

Gross Pathology

The animals were examined by pathologists according to a standardized protocol. They were first weighed, and the average weight of the different cohorts defined hereafter was compared by using the Mann-Whitney test. To better reflect the diversity of clinical presentations, these calves were divided into 4 categories according to the severity of their musculoskeletal deformities (Technical Appendix 3, Figure 1, http://wwwnc.cdc.gov/EID/article/20/8/12-1890-Techapp3.pdf). Animals with neurologic signs and apparently normal body shape were given a whole-body deformity (WBD) score of 0. Those with altered body shape were scored 1, 2, or 3 depending on whether 1, 2 or 3 skeletal segments were deformed, respectively (spine, forelimbs, or hind limbs). In addition, the maximum widths of the foramen magnum and of the spinal cord at that level were measured. The occipital ratio reported is the result of dividing the second width by the first. After a thorough examination, the following organs

were sampled: brain, spinal cord, lung, myocardium, thymus, liver, spleen, kidney, duodenum, jejunum, ileum, colon, and skeletal muscles. From the brain, specimens from 6 distinct regions were sampled: olfactory bulb (paleopallium), cortex (neopallium), diencephalon, midbrain (mesencephalon), cerebellum, and pons (metencephalon). The spinal cord samples consisted of the segment corresponding to the fourth cervical vertebra. Nine skeletal muscles were sampled, 2 spinal muscles (semispinalis capitis and longissimus thoracis), 3 forelimb muscles (supraspinatus, extensor carpi radialis, and flexor carpi ulnaris), and 3 hind limb muscles (semimembranosus, quadriceps femoris, and peroneus tertius). For the semispinalis capitis, a piece of muscle was taken directly below the fourth cervical vertebra from either side of the pathologic curvature of the spine. Each tissue sample was then divided in 2 fragments: 1 was frozen at –80°C (for subsequent RNA extraction), and the other was immersed in 10% buffered formalin (histopathology). Postmortem serum was extracted from the cardiac ventricles and kept frozen at –20°C until use.

Histology

All tissues were routinely processed in paraffin wax, cut at 4-µm thickness, mounted on glass slides, deparaffinized in xylene, dehydrated in ethanol, and stained with hematoxylin and eosin. Luxol fast blue staining was chosen for visualizing myelin in spinal cord and efferent axons in ventral roots. Perls' acid ferrocyanide reaction was used to reveal iron compounds in the ferric state, with nuclear fast red for counterstaining. The control cohort included five 1-day-old calves that were simultaneously negative for viral RNA and Schmallenberg virus (SBV) IgG. The extent of histologic changes was reported semiquantitatively by using a score of 0, 1, 2, or 3 depending on whether the histologically normal tissue was 100%, 75%–100%, 25%–75%, or <25% of the area examined.

Detection of SBV-specific Antibodies

IgG against the nucleoprotein of SBV was detected in postmortem serum by using the ELISA kit (ID Screen Schmallenberg virus Indirect v.1) recently made available by ID.vet Innovative Diagnostics (ID.vet Innovative Diagnostics, Montpellier, France). In this assay, results are expressed as percentages of the reference signal yielded by a positive control serum, with serologic status defined by the manufacturer as negative (≤60%), doubtful (>60% and ≤70%) or positive (>70%). Positive and negative reference bovine serum was drawn within a previously archived bank (*1*).

Detection of Viral RNA

The virus genome was sought in the tissues of all animals belonging to ≥1 of the following categories: 1) calves with reported neurologic signs before death, 2) calves displaying musculoskeletal deformities, and 3) calves that died spontaneously soon after birth and in which no unequivocal cause of death was identified at necropsy. Tissue samples collected in the necropsy room were homogenized (Qiagen TissueLyser, Venlo, the Netherlands, 30 Hz for 2 min) in TRIzol reagent (Invitrogen, Merelbeke, Belgium), and total RNA was extracted from the resulting homogenate according to the manufacturer's instructions. Precipitated RNA was resuspended in 10% dimethyl sulfoxide (Sigma-Aldrich, Diegem, Belgium) and stored at –80°C until use. A Mastercycler pro thermocycler (Eppendorf, Rotselaar, Belgium) and a StepOnePlus real-time PCR system (Applied Biosystems, Gent, Belgium) were used for reverse transcription (RT) and quantitative PCR (qPCR), respectively. Detection of the SBV genome was performed according to Bilk et al. (*2*) and that of the bovine viral diarrhea virus (BVDV) genome according to La Rocca and Sandvik (*3*), except that the heterologous internal control (β-actin) and each virus were detected separately. Briefly, the 3 RT-qPCRs were performed with the AgPath-ID One-Step RT-PCR kit (Applied Biosystems) by using a total volume of 25 μL. Primer and probes used in this study are listed in the Technical Appendix 1 Table). A master mix consisting of 4.5 μL RNase-free water, 12.5 μL 2 × RT-PCR buffer, 1.0 μL 25 × RT-PCR enzyme mix, 2.0 μL SBV-specific (BVDV- or β-actin-specific) primer–probe mix (10 μM target-specific primers + 1.875 μM target-specific probe) for 1 reaction, and 5 μL RNA template was added. For amplification of SBV RNA, the following temperature profile was used: 10 min at 45°C (RT), 10 min at 95°C (inactivation of RT and activation of Taq polymerase), followed by 42 cycles of 15 s at 95°C (denaturation), 20 s at 56°C (annealing), and 30 s at 72°C (elongation). For amplification of BVDV RNA, the temperature profile was the following: 10 min at 50°C, 2 min at 95°C, and 40 cycles of 15 s at 95°°C (denaturation) and 30 s at 60°C (combined annealing and elongation). The detection of the bluetongue virus genome was performed according to Toussaint et al. (*4*) with slight modifications. Briefly, a pan-BTV 2-step RT-qPCR targeting genome segment-5 of bluetongue viruses was carried out, again with β-actin mRNA as internal control. The Gene Expression Master Mix (Applied Biosystems) was used for a first qPCR with the following conditions: 10 min at 95°C, then 45 cycles of 15 s at 95°C (denaturation) and 1 min at 58°C (combined annealing and elongation). The cutoff was set at 43 as determined by dilutions of the synthetic RNA controls according to Toussaint et al. (*4*). Positive and doubtful samples are then theoretically submitted to a segment-1 or a segment-7–specific

PCR as described in Garigliany et al. (*5*). Because the step-1 qPCR did not detect any positive or doubtful samples from the tissues examined here, the step 2 was not necessary. For the detection of the genetic material of the 3 viruses targeted, all reactions were carried out twice. In 90% of cases, the results were identical. Whenever the results were conflicting, a third RT-qPCR was performed, and the majority result was taken as definitive. A negative extraction control, and both a negative and a positive RT and amplification controls, were always included.

Refuting Idiopathic Arthrogryposis

The cause of the congenital arthrogryposis syndrome affecting the local Belgian Blue cattle population was recently assigned to a focal transversion in the splice acceptor region of the phosphatidylinositol glycan anchor biosynthesis class H (PIGH) intron 1. All animals were genotyped to exclude the possibility that this mutation contributed to the observed lesions using the 5′ exonuclease diagnostic assay recently made available (Sartelet A et al., unpub. data).

References

1. Garigliany MM, Bayrou C, Kleijnen D, Cassart D, Desmecht D. Schmallenberg virus in domestic cattle, Belgium, 2012. Emerg Infect Dis. 2012;18:1512–4. PubMed http://dx.doi.org/10.3201/eid1809.120716

2. Bilk S, Schulze C, Fischer M, Beer M, Hlinak A, Hoffmann B. Organ distribution of Schmallenberg virus RNA in malformed newborns. Vet Microbiol. 2012;159:236–8. PubMed http://dx.doi.org/10.1016/j.vetmic.2012.03.035

3. La Rocca SA, Sandvik T. A short target real-time RT-PCR assay for detection of pestiviruses infecting cattle. J Virol Methods. 2009;161:122–7. PubMed http://dx.doi.org/10.1016/j.jviromet.2009.06.005

4. Toussaint JF, Sailleau C, Breard E, Zientara S, De Clercq K. Bluetongue virus detection by two real-time RT-qPCRs targeting two different genomic segments. J Virol Methods. 2007;140:115–23. PubMed http://dx.doi.org/10.1016/j.jviromet.2006.11.007

5. Garigliany M, De Leeuw I, Kleijnen D, Vandenbussche F, Callens J, Van Loo H, et al. The presence of bluetongue virus serotype 8 RNA in Belgian cattle since 2008. Transbound Emerg Dis. 2011;58:503–9. PubMed http://dx.doi.org/10.1111/j.1865-1682.2011.01230.x

Technical Appendix Table. Primers and probes

Target	Primer/probe name	Sequence (5′→3′)†	Genome position
SBV	382-F	TCA GAT TGT CAT GCC CCT TGC	382–402‡
	469-R	TTC GGC CCC AGG TGC AAA TC	450–469‡
	P-408	FAM-TTA AGG GAT GCA CCT GGG CCG ATG GT-BHQ1	408–433‡
BVDV	106-F	CCA TGC CCT TAG TAG GAC TAG C	106–127§
	190-R	GCG TCG AAC CAC TGA CGA CT	190–209§
	P-162	FAM-TGG ATG GCT TAA GCC CTG AGT ACA G-EDQ	162–186§
BTV	F_1–19	GGC AAC YAC CAA ACA TGG A	1–19¶
	R_76–57	AAA GTY CTC GTG GCA TTW GC	57–76¶
	P_49–27	FAM-CYC CAC TGA TRT TGT ATT TTC TCA A-TAMRA	49–27¶
β-actin	F_1005–1029	CAG CAC AAT GAA GAT CAA GAT CAT C	1005–1029
	R_1135–1114	CGG ACT CAT CGT ACT CCT GCT TT	1114–1135
	P_1081–1105	FAM-TCG CTG TCC ACC TTC CAG CAG ATG T-TAMRA	1081–1105

*SBV, Schmallenberg virus; BVDV, bovine viral diarrhea virus; BTV, bluetongue viruses; FAM = 6-carboxyfluorescein; EDQ, Eclipse Dark Quencher; BHQ1, Black Hole Quencher-1.
†Reverse primers complementary to positive-sense target strand.
‡n the SBV genome segment-S (GenBank accession no. HE649914).
§In the BVDV NADL genome (GenBank accession no. M31182)
¶In the BTV genome segment-5.
