# Supplementary material for: Natural Intrauterine Infection with Schmallenberg Virus in Malformed Newborn Calves
Source: Emerg Infect Dis. 2014 Aug;20(8):1327–30. doi: 10.3201/eid2008.121890 (PMC4111192; doi:10.3201/eid2008.121890)
Supplement: Technical Appendix 2 — Gross pathology and histology. [file 12-1890-Techapp-s2.pdf]

Article DOI: http://dx.doi.org/10.3201/eid2008.121890

Natural Intrauterine Infection with Schmallenberg Virus in Malformed Newborn Calves

Technical Appendix 2

Gross Pathology and Histology

The Body as a Whole

Schmallenberg virus (SBV)–positive calves were significantly lighter than SBV-negative calves (32 ± 15 vs. 49 ± 4 kg, $p<0.05$). The large deviation between SBV cases denotes a heterogeneous deficit of body weight. Notably, there was an obvious correlation between the body mass deficit and the severity of deformations in whole-body conformation (Table 1). Also, most deformed animals died spontaneously within 24 hours after birth (scores whole-body deformity score [WBD]–2 and –3). Among the least affected, the majority was euthanized. No significant morphologic changes were noted in the thoracic and abdominal cavities and organs.

The Axial Musculoskeletal System

Overall, permanent deviations of the axial skeleton in all 3 planes were observed (Technical Appendix 3 Figures 1, 2, http://wwwnc.cdc.gov/EID/article/20/8/12-1890-Techapp3.pdf). The most common observation (11/15) was a lateral deviation of the cervical spine (torticollis). In 2 cases (WBD-2), lateral deviations of the thoracic segment of the spine was noticed. In addition, in the most distorted animals (WBD-3), the torticollis was accompanied by a dorso-ventral deviation of the thoracolumbar spine (either kyphosis or lordosis) and by a gradual rotation of the column itself, giving it a helical conformation. In the most severe cases (WBD-3), the vertebrae displayed deformities, with distortions in the shape of the spinal canal but without stenosis. Besides, deficits in muscle mass in the spinal muscles were observed in all cases. Regarding their magnitude and distribution, no consistent pattern was identified. These changes seemed consistent with prenatal occurrence of processes leading to hypoplasia and/or atrophy. In addition, after section, muscles revealed whitish and multifocally distributed areas.

The Appendicular Musculoskeletal System

Most cases (13/15) displayed joint fixation of 1 or all joints of ≥1 limbs, which is referred to as arthrogryposis. Joint involvement was always symmetric (right/left ratio = 1), whereas tetramelic involvement was observed in the most severe cases only (WBD-3). When a single pair of limbs was involved, it almost always was the forelimbs. The joints were fixed either in hyperflexion (66% of affected joints) or in hyperextension. Section of the tendons always restored the mobility of the corresponding joint, and articular cavities were filled with a normal yellow viscous synovial fluid. Tendons spanning fixed joints were shorter than expected, and corresponding muscles displayed altered mass (decreased) and color (multifocal whitish patches). On visual inspection, bone lengths remained proportional to the size of the calves, but some articular surfaces deviated from the plane expected.

The Head

The general shape of the head was often altered, but the magnitude of the changes was not correlated with axial and appendicular musculoskeletal changes (Technical Appendix 3 Figure 3). Two calves displayed a horse-like (long nose and reduced height of cranial cavity) and 2 others a pig-like head (increased height and reduced length), 4 calves were brachygnathic (upper teeth protruding beyond lower teeth) and 1 prognathic (lower jaw too far forward). Notably, the head of a third of the calves displayed diverging sagittal axes, with an angle between the sagittal plane of the upper and lower halves of the head (Technical Appendix 3 Figure 3). There was no evidence of muscle injury or mandibular arthritis.

The Central Nervous System

Major alterations were systematically observed after the skull and spinal canal were opened (Figure 1; Technical Appendix 3 Figure 4). These changes involved the spinal cord and the telencephalon, whereas the brainstem (medulla oblongata, pons, mesencephalon, and diencephalon) and cerebellum were intact (except 1 hypoplastic cerebellum). In WBD-2 and –3 cases, a decrease in the cross-sectional area of the spinal cord was observed over its entire length (Technical Appendix 3 Figure 4). Because the width of the spinal canal remained similar to that of healthy calves, a large space filled with cerebrospinal fluid (CSF) was always interposed between the spinal cord and most of the canal circumference. In addition, the general structure of the spinal cord was preserved, with an intact ventral median fissure and a normally proportioned central canal. These changes are referred to as micromyelia without myelodysplasia and seem consistent with prenatal occurrence of processes leading to hypoplasia and/or atrophy. Remarkably, the magnitude of the decrease in spinal cord cross-

sectional area, as measured by the occipital ratio, correlated positively with the severity of axial and appendicular musculoskeletal deformities (Technical Appendix 3, Figure 5). Moreover, the neopallial part of the telencephalon was nearly completely absent in most SBV cases, whereas the paleopallium-olfactory components, the archipallium-hippocampus, fornix and basal nuclei were comparatively spared (Figure 1). The amount of CSF was consistently increased due to the filling of the space normally occupied by the neopallium. These macroscopic alterations produced 3 distinct morphotypes. In some calves, multiple, bilateral, and randomly located cystic cavities were detected in the neopallium, most of which communicated with the ipsilateral ventricle. These alterations were referred to as porencephaly. In other cases, all that remained from the neopallium was a thin, nearly transparent membrane with sometimes a few floating smooth-surfaced islets resembling cortex. The membrane was kept distended by the CSF that filled the space normally occupied by cerebral tissue. These changes were referred to as hydranencephaly. Finally, in a third subset of cases, the brain appeared normal after the skull was opened but revealed a severe, bilateral and symmetrical dilatation of lateral ventricles after section.

Histopathology

Brain

No significant alterations were seen in the brainstem and cerebellum (Technical Appendix 4 Table 2). In particular, the neuron number was not reduced; there was no perivascular cuffing of lymphocytes, no hemorrhages, no hemosiderin-laden macrophages, no mineralization, no gliosis, nor stigmata of periependymal inflammation. In the telencephalon, only minor changes were seen. In porencephalic cases, the cavities were lined by astrocytes and surrounded by occasional foci of gliosis. The membrane typical of hydranencephaly consisted mostly of astroglia, pia mater, blood vessels, and sometimes a few inflammatory cells. The thinned neopallium accompanying hydrocephaly was not remarkable either, again showing evenly distributed gliosis.

Spinal Cord

Examination of the spinal cord by light microscopy instantly revealed a very significant decrease in the neuron numbers along its entire length (Table 2). Conversely, like the encephalon, the presence of changes characteristic of recent or past necrosis or inflammation was not obvious. There was no perivascular cuffing of lymphocytes, no mineralization, no hemorrhages, no hemosiderin-laden macrophages. Some foci of gliosis and images consistent of neuronophagia were observed, but they remained very rare. In the most deformed animals

(WBD-3), there were no motor neuron left in the gray matter and no efferent axons in the ventral horns (Figure 2). Generally, the deficit in motor neurons was more pronounced than the deficit in sensory neurons (Table 2). Finally, there was an inverse correlation between the number of spinal neurons and the magnitude of whole-body deformation (Table 2).

Muscles

Apart in muscle parts injured by prolonged decubitus, changes characteristic of ongoing necrotizing or inflammatory processes were absent. In particular, hypercontraction bands, myofibers containing eosinophilic coagulum, leukocytic invasion and mineralization were absent. Conversely, muscle sections consistently displayed a diffuse pattern of increased fiber size variation with connective and adipose tissue infiltrations (Technical Appendix 3 Figure 6; Technical Appendix 4 Table 1). Large-diameter fibers were admixed with severely atrophied fibers, and islets of muscle-like tissue were dissected from each other by variable size areas where myofibers were replaced by fibrous connective and adipose tissues. In addition, myotubes were consistently observed in most-altered muscles examined, suggesting widespread attempts of regeneration and repair. Although the nature of the morphologic changes was the same throughout the muscles examined, their magnitude, as judged from the muscle/connective tissue ratio, varied from place to place which explains the scattered whitish plaques observed macroscopically.

Other Organs

No reproducible lesion was observed in other thoracic or abdominal organs. A lymphocytic infiltration of the small intestine submucosa was noticed in the majority of SBV cases, hepatocytes swelling, increased granularity and eosinophilia in 6 cases and a lymphocytic interstitial pneumonia in 5 cases (Technical Appendix 4 Table 3).
