# Supplementary material for: Natural Intrauterine Infection with Schmallenberg Virus in Malformed Newborn Calves
Source: Emerg Infect Dis. 2014 Aug;20(8):1327–30. doi: 10.3201/eid2008.121890 (PMC4111192; doi:10.3201/eid2008.121890)
Supplement: Technical Appendix 3 — Definition of whole-body deformity (WBD) scores in Schmallenberg virus (SBV)–infected newborn calves, Belgium, 2012; deformities of the axial skeleton ; deformities of the head ; micromyelia ; occipital ratios (mean ± SD) in control and SBV-positive calves ; and end-stage muscles in a typical SBV-infected calf. [file 12-1890-Techapp-s3.pdf]

Article DOI: http://dx.doi.org/10.3201/eid2008.121890

Natural Intrauterine Infection with Schmallenberg Virus in Malformed Newborn Calves

Technical Appendix 3

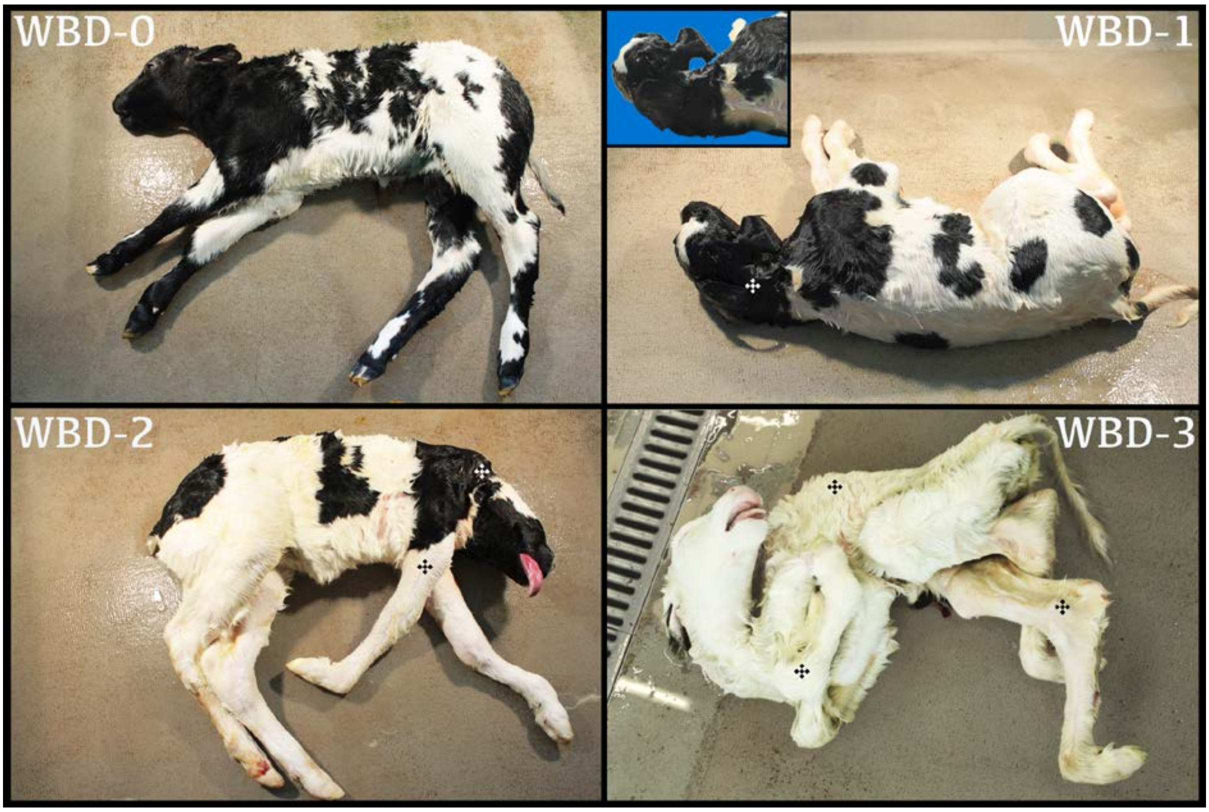

Technical Appendix Figure 1. Definition of whole-body deformity (WBD) scores in Schmallenberg virus (SBV)–infected newborn calves, Belgium, 2012. Animals with neurologic signs and apparently normal body shape were given a WBD score of 0. Those with altered body shape were scored 1, 2, or 3 depending on whether 1, 2 or 3 skeletal segments were deformed, respectively (spine, forelimbs, or hind limbs). Marks: deformed skeletal segments.

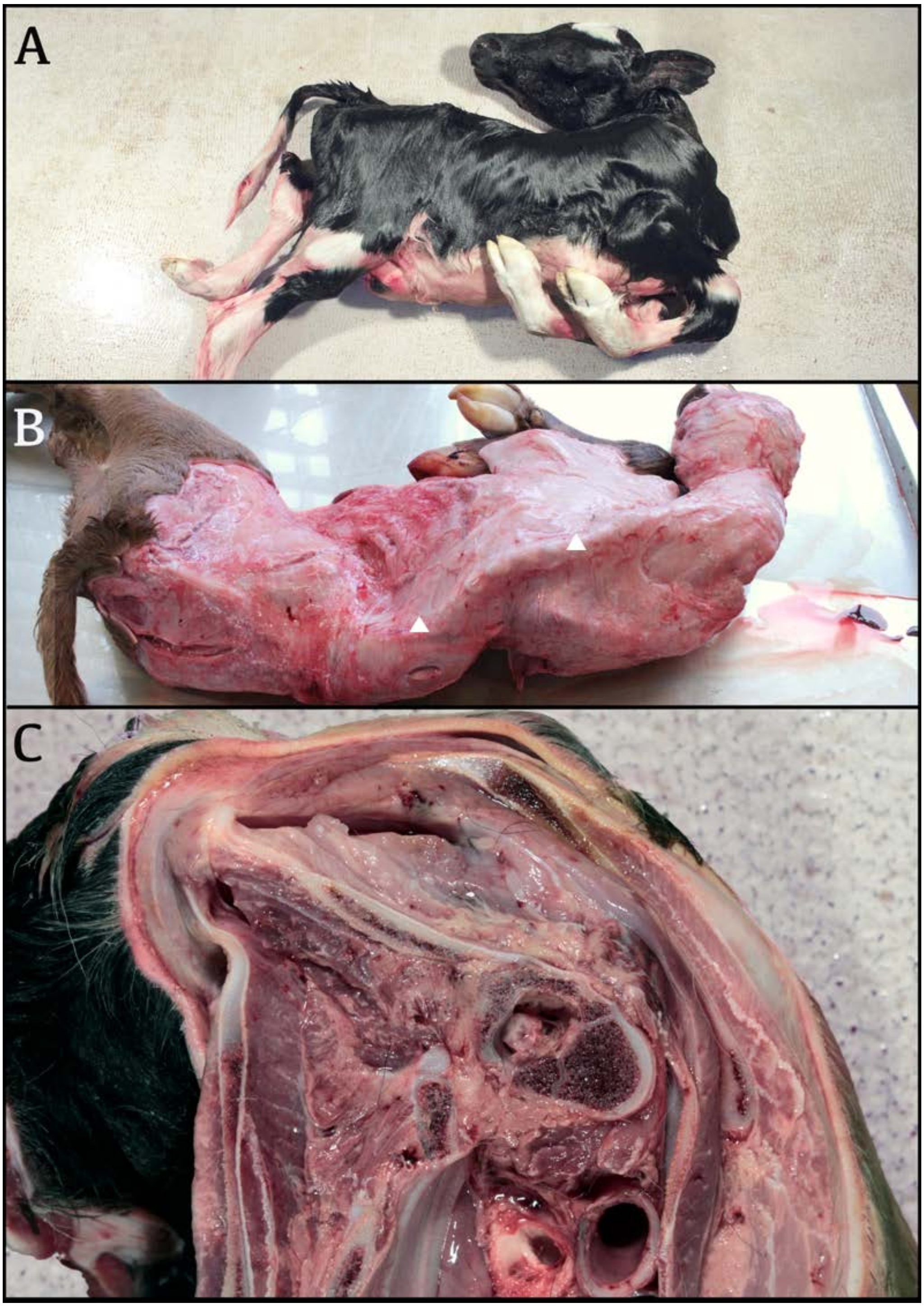

Technical Appendix Figure 2. Deformities of the axial skeleton in Schmallenberg virus–infected calves. A) Torticollis. B) Lateral deviations and asymmetric loss of musculus longissimus dorsi volume (compare left and right muscle mass at the triangles). C) Loss of volume and discoloration of right cervical muscles.

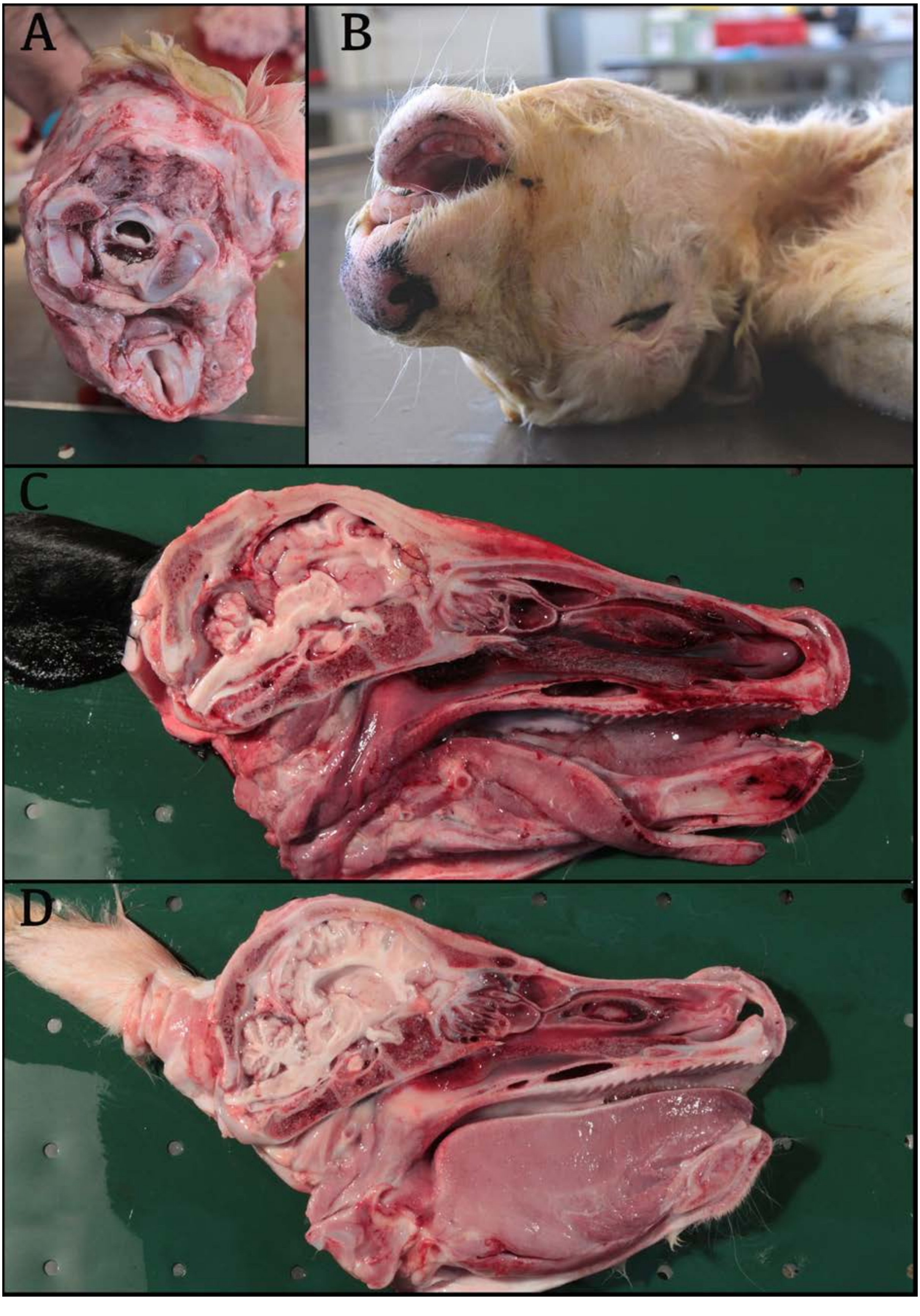

Technical Appendix Figure 3. Deformities of the head in Schmallenberg virus–infected calves. A) Diverging sagittal axes. B) Brachygnathism. Horse-like (C) and pig-like (D) profiles.

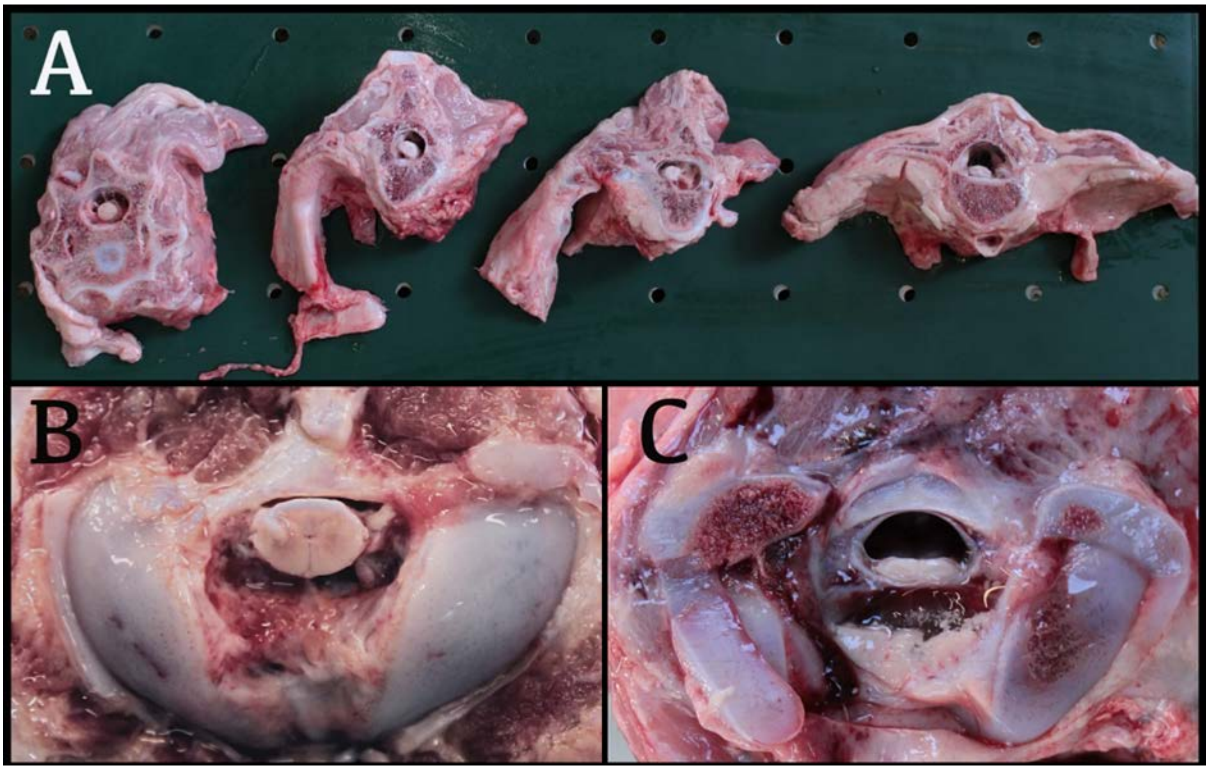

Technical Appendix 4. Micromyelia in Schmallenberg virus–infected calves. A) Successive sections showing micromyelia extending over the entire length of the spinal cord. B) Cross-section of spinal cord when it emerges from the foramen magnum in a control calf. C) The same in an SBV-positive calf.

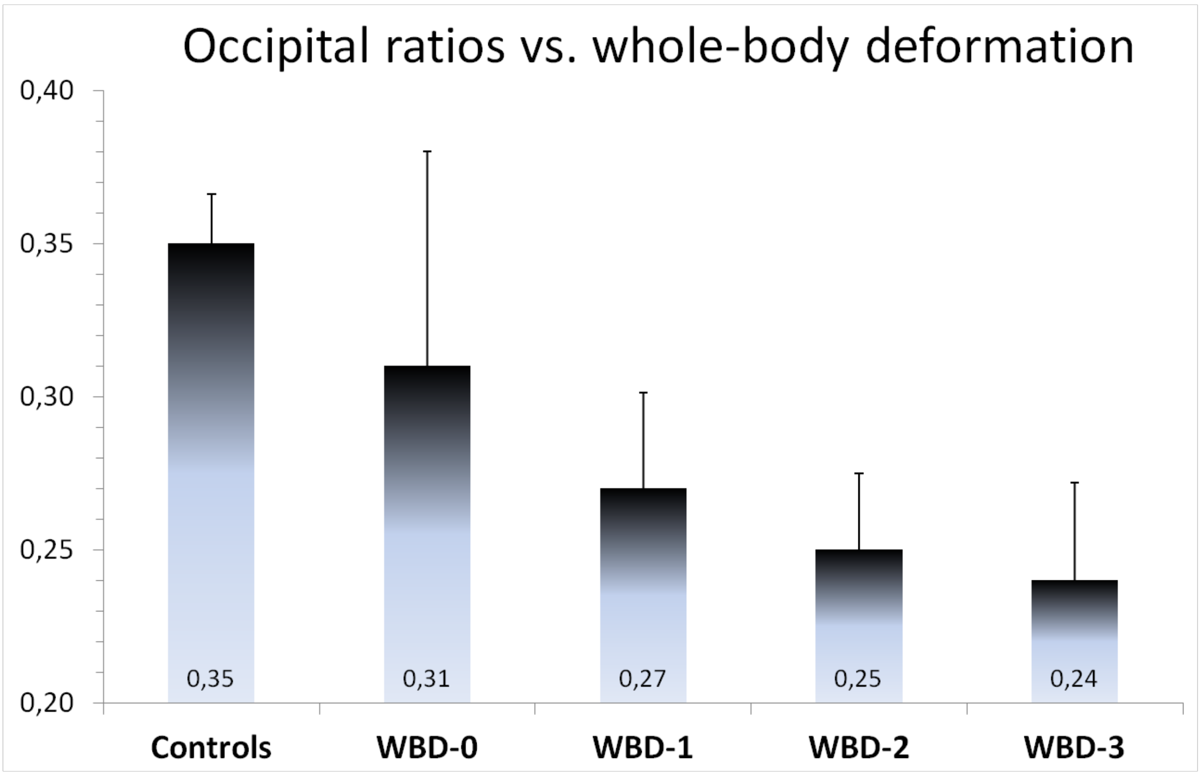

Technical Appendix 5. Occipital ratios (mean ± SD) in control and Schmallenberg virus–positive calves. Note negative correlation with whole-body deformity scores. The maximum width of the foramen magnum and of the spinal cord at that level were measured. The occipital ratio of a specific calf is the result of dividing the second width by the first.

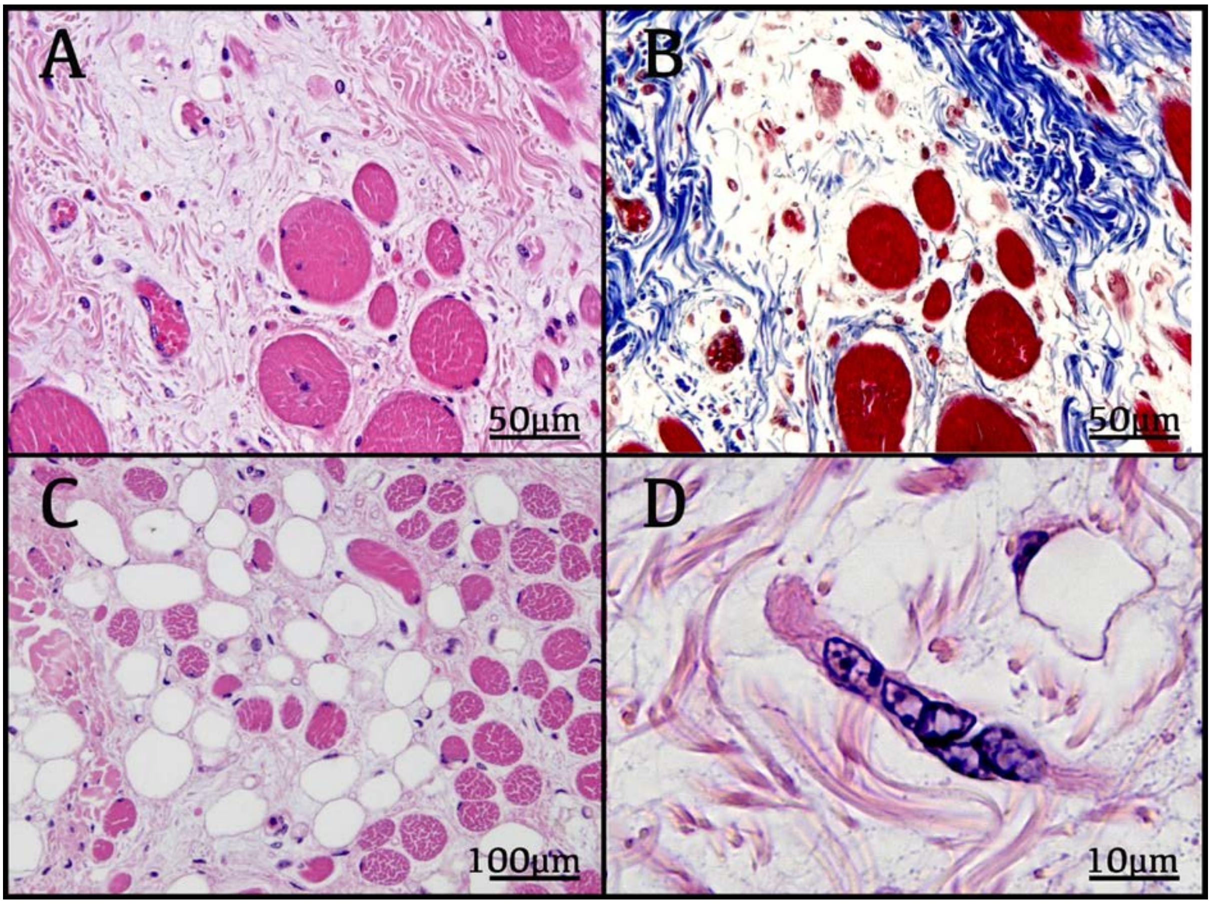

Technical Appendix 6. End-stage muscles in a typical Schmallenberg virus–infected calf. Age- and site-matched histologic sections of semispinalis capitis muscle. A) Large-diameter fibers were admixed with severely atrophied fibers, and islets of muscle-like tissue were dissected from each other by variable size areas where myofibers were replaced by fibrous connective tissue (B) and by fat (C). D) A myotube suggesting an attempted regeneration. Hematoxylin and eosin (A, C, D) and Masson's trichrome (B) stains. Original magnification ×100 (C), ×200 (A, B), and ×1,000 (D).
