# Supplementary material for: Natural Intrauterine Infection with Schmallenberg Virus in Malformed Newborn Calves
Source: Emerg Infect Dis. 2014 Aug;20(8):1327–30. doi: 10.3201/eid2008.121890 (PMC4111192; doi:10.3201/eid2008.121890)
Supplement: Technical Appendix 4 — Distribution of microscopic lesions and virus-specific RNA in the skeletal mucles of 15 Schmallenberg virus–infected newborn calves ; distribution of microscopic lesions and virus-specific RNA in the central nervous system ; and distribution of microscopic lesions and virus-specific RNA in thoraco-abdominal organs. [file 12-1890-Techapp-s4.pdf]

Article DOI: http://dx.doi.org/10.3201/eid2008.121890

Natural Intrauterine Infection with Schmallenberg Virus in Malformed Newborn Calves

Technical Appendix 4

Technical Appendix Table 1. Distribution of microscopic lesions and virus-specific RNA in the skeletal muscles of 15 SBV-infected newborn calves*

	WBD/calf ID														
	WBD-0		WBD-1				WBD-2				WBD-3				
Skeletal muscle	A	B	C	D	E	F	G	H	I	J	K	L	M	N	O
Spinal muscles															
Musculus semispinalis capitis, cas															
RT-qPCR	NT		–	–	–	–	–	–	NT	–	–	–	–	–	–
Histology	NT	0	0	0	3	0	3	3	NT	3	3	3	3	3	3
Musculus semispinalis capitis, ces															
RT-qPCR	NT	–	–	–	–	–	–	–	NT	–	–	–	–	–	–
Histology	NT	1	0	0	2	0	3	3	NT	3	3	3	3	3	3
Musculus longissimus thoracis															
RT-qPCR	NT	–	–	–	–	–	–	–	NT	–	–	–	–	–	–
Histology	NT	0	0	0	0	0	0	0	NT	0	2	3	3	1	2
Forelimb muscles															
Musculus supraspinatus															
RT-qPCR	NT	–	–	–	+	–	–	–	NT	–	–	–	–	–	–
Histology	NT	0	0	0	0	0	0	0	NT	0	2	3	3	1	2
Musculus extensor carpi radialis															
RT-qPCR	NT	–	–	–	+	–	–	–	NT	–	–	–	–	–	–
Histology	NT	1	0	0	0	0	0	0	NT	1	3	3	3	0	3
Musculus flexor carpi ulnaris															
RT-qPCR	NT	–	–	–	+	–	–	–	NT	–	–	–	–	–	–
Histology	NT	1	0	0	1	0	0	0	NT	3	3	3	3	2	3
Hind limb muscles															
Musculus semimembranosus															
RT-qPCR	NT	–	–	–	+	–	–	–	NT	–	–	–	–	–	–
Histology	NT	0	1	0	0	0	0	3	NT	3	3	3	3	3	3
Musculus quadriceps femoris															
RT-qPCR	NT	–	–	–	+	–	–	–	NT	–	–	–	–	–	–
Histology	NT	0	0	0	1	0	3	3	NT	3	3	3	3	3	3
Musculus peroneus tertius															
RT-qPCR	NT	–	–	–	+	–	–	–	NT	–	–	–	–	–	–
Histology	NT	1	1	0	1	1	2	2	NT	0	3	3	3	3	3

*Extent of histologic changes was reported semiquantitatively by using a score of 0, 1, 2 or 3 depending on whether the histologically normal tissue extended over 100%, 75%–100%, 25%–75%, or <25% of the area examined and intensity of shading reflects this. SBV, Schmallenberg virus; WBD, whole-body deformity score; cas, concave side; RT-qPCR, reverse transcription quantitative PCR; NT, not tested; ces, convex side.

Technical Appendix Table 2. Distribution of microscopic lesions and virus-specific RNA in the CNS of 15 SBV-infected newborn calves

	WBD/calf ID														
	WBD-0		WBD-1				WBD-2				WBD-3				
CNS portion	A	B	C	D	E	F	G	H	I	J	K	L	M	N	O
Paleopallium															
RT-qPCR	NT	+	NT	–	+	+	–	–	NT	–	+	+	–	–	–
Histology	NT	GL	0	0	GL	GL	0	0	NT	0	0	0	0	0	0
Neopallium															
RT-qPCR	+	+	–	+	+	+	+	+	+	+	+	+	–	+	+
Histology	0	GL	0	G	GL	0	0	0	NT	GL	0	0	0	0	0
Diencephalon															
RT-qPCR	NT	+	NT	–	+	+	+	–	NT	–	+	+	–	+	–
Histology	NT	0	0	GL	GL	0	0	0	NT	0	GL	0	0	0	0
Mesencephalon															
RT-qPCR	NT	+	NT	–	+	+	+	+	NT	+	+	+	+	+	–
Histology	0	0	0	0	0	0	0	0	NT	0	GL	0	0	0	0
Cerebellum															
RT-qPCR	NT	+	NT	–	+	+	–	+	NT	–	–	+	–	–	–
Histology	0	0	0	ED	0	0	0	0	NT	0	0	0	0	0	0
Pons															
RT-qPCR	NT	+	NT	–	+	+	+	+	NT	–	+	+	–	+	–
Histology	0	0	0	ED	0	0	ED	0	NT	0	ED	0	0	0	0
Spinal cord†															
RT-qPCR	NT	+	+	+	+	+	+	+	+	+	+	+	–	+	+
Histology	NT	0	0	ED	0	0	0	ED	NT	ED	ED	ED	ED	0	ED

*CNS, central nervous system; SBV, Schmallenberg virus; WBD, whole-body deformity score; 0, no lesion; RT-qPCR, reverse transcription quantitative PCR; +, positive; –, negative; GL, gliosis; ED, edema;
†Sections were made at the level of the fourth cervical vertebra.

Technical Appendix Table 3. Distribution of microscopic lesions and virus-specific RNA in thoraco-abdominal organs of 15 SBV-infected newborn calves*

	WBD/calf ID														
	WBD-0		WBD-1				WBD-2				WBD-3				
Organ	A	B	C	D	E	F	G	H	I	J	K	L	M	N	O
Thorax															
Lung															
RT-qPCR	–	–	–	+	+	+	+	–	–	–	–	–	–	+	–
Histology		IP		IP		IP			NT		NT			IP	
Thymus															
RT-qPCR	NT	–	–	–	–	–	–	–	–	–	–	–	–	–	–
Histology	NT								NT		NT				
Myocardium															
RT-qPCR	NT	–	–	–	+	–	–	–	–	–	–	–	–	–	–
Histology	NT								NT		NT				
Abdomen															
Duodenum															
RT-qPCR	NT	–	–	–	–	–	–	–	–	–	–	–	–	–	–
Histology	NT								NT		NT			HL	
Jejunum															
RT-qPCR	–	–	–	–	–	–	–	–	–	–	–	–	–	–	–
Histology	LI			HL					NT	LI	NT		HL	HL	HL
Ileum															
RT-qPCR	–	–	–	–	–	–	–	–	–	–	–	–	–	–	–
Histology	HL			HL	HL	HL	HL	HL	NT	HL	NT		HL	HL	HL
Colon	NT	–	–	+	–	+	–	–	–	–	–	–	–	–	–
RT-qPCR															
Histology	NT								NT		NT				
Kidney															
RT-qPCR	–	–	–	–	+	–	–	–	–	–	–	–	–	–	–
Histology									NT		NT				
Liver															
RT-qPCR	–	–	–	–	+	–	–	–	–	–	–	–	–	–	–
Histology		DEG		DEG		DEG		DEG	NT		NT	DEG	CON	DEG	
Spleen															
RT-qPCR	–	–	–	–	+	–	–	–	–	–	–	–	–	–	–
Histology									NT		NT		CON		

SBV, Schmallenberg virus; WBD, whole-body deformity score; RT-qPCR, reverse transcription quantitative PCR; NT, not tested; +, positive; –, negative. Histologic alterations are as follows: CON, congestion; DEG, degeneration; LI, leukocytic infiltration; IP, interstitial pneumonia. HL, lymphoid hyperplasia. Blank spaces denotes absence of alteration.
