# Supplementary material for: Natural Intrauterine Infection with Schmallenberg Virus in Malformed Newborn Calves
Source: Emerg Infect Dis. 2014 Aug;20(8):1327–30. doi: 10.3201/eid2008.121890 (PMC4111192; doi:10.3201/eid2008.121890)
Supplement: Technical Appendix 5 — Diagnostic considerations. [file 12-1890-Techapp-s5.pdf]

Article DOI: http://dx.doi.org/10.3201/eid2008.121890

Natural Intrauterine Infection with Schmallenberg Virus in Malformed Newborn Calves

Technical Appendix 5

Diagnostic Considerations

Virus Detection

To our knowledge, there is currently 1 well-documented source with which to compare the rate of detection of Schmallenberg virus (SBV) RNA in suspected SBV cases (*1*). At first sight, these detection rates appear very different. This is, however, not surprising because the definition of a suspected SBV case is very different between the 2 studies. For De Regge et al., a suspected case was an aborted calf with the typical malformations described for SBV infection: arthrogryposis, hydranencephaly, or hypoplasia of cerebrum and/or cerebellum (*1*). With this definition, 38% of the suspected cases proved SBV RNA positive. In the present study, we considered that, in spring 2012, the whole spectrum of SBV-related conditions in the bovine species was, by definition for a disease that had emerged 2 months before, unknown. This is the reason we opted for a broader definition of suspected SBV cases: 1) calves with neurologic signs after death or 2) calves with musculoskeletal deformities or 3) calves in which no unequivocal cause of death was identified at necropsy. By using this broader definition, 22% of suspected cases proved SBV RNA positive. Further, if we restrict the cohort of suspected SBV cases to those compatible with the afore-described narrow definition, we then had 15 suspected cases in total, of which 13 proved SBV RNA positive, thus a detection rate of 87%. In this instance, the starting cohort is similar, except that aborted calves were tested on the one hand (*1*), and calves born at term on the other (this study). Considering the methodologic aspects, it seems that the higher rates we observed could be due to 1 difference or to the combination of 3 differences, each with increasing sensitivity. First, we stored the tissue samples at –80°C often within 24 h after death and, in all cases, within 48 h, which is favorable to conserving viral RNA. Second, the extraction procedure implemented in this study (TRIzol) is deemed more efficient than using commercially available kits. Third, whereas SBV and β-actin RNAs were amplified together in a duplex

assay in the first study (*1*), we amplified SBV RNA and β-actin RNA separately, which, in our hands, results in a higher sensitivity than do duplex assays.

To our knowledge, this study is the most comprehensive so far in terms of distribution of SBV RNA in the calf naturally infected in utero (26 sites were tested, cf. Technical Appendix 4 Tables 1–3). It clearly appears that testing the spinal cord at C4 maximizes the probability of detecting the virus (93%). The viral RNA is also often present in the neopallium (87%) and the mesencephalon (83%). Taking a fragment at each of these 3 sites and pooling them for reverse transcription quantitative PCR can detect all, which confirms recently published reports (*1*,*2*). For cons, the paleopallium, diencephalon, and cerebellum seem definitely not suitable for diagnosis. Furthermore, we show that the lymphoid and related organs (spleen, thymus, liver) contain the virus only very rarely (1 of 15 calves), which suggests that SBV is not lymphotropic. Finally, the screening of the organs revealed that the virus is quite frequently detectable in the lungs (one third of cases). This result is surprising because, unlike the CNS, lungs are exposed to circulating antibodies. This observation may reflect an important aspect of the biology of the new virus in its ruminant host.

Field Diagnosis

The data suggest that, in the field and when resources are limited, relying on precolostral positive serology to assign the cause of a congenital triad por-/hydranencephaly-micromyelia-arthrogryposis to SBV is rarely wrong. This conclusion is obviously valid only in areas where phylogenetically related viruses, those likely to cause cross-reactions, are absent. In the present context, the cause of all cases of arthogryposis undergoing necropsy during January 1–June 30, 2012, was established: either the SBV genetic material or SBV IgGs were detected, or the animal was homozygous for the *PIGH* mutation. The presence of this genetic defect in a population does not, however, hamper the differential diagnosis in field situations: the genetically determined arthrogryposis syndrome does not include por-/hydranencephaly or micromyelia. Further, the calves concerned always displayed a cleft palate, which was never observed among SBV cases.

References

1. De Regge N, van den Berg T, Georges L, Cay B. Diagnosis of Schmallenberg virus infection in malformed lambs and calves and first indications for virus clearance in the fetus. Vet Microbiol. 2013;162:595–600. PubMed http://dx.doi.org/10.1016/j.vetmic.2012.11.029

2. Bilk S, Schulze C, Fischer M, Beer M, Hlinak A, Hoffmann B. Organ distribution of Schmallenberg virus RNA in malformed newborns. Vet Microbiol. 2012;159:236–8. PubMed http://dx.doi.org/10.1016/j.vetmic.2012.03.035
