# Supplementary material for: Natural Intrauterine Infection with Schmallenberg Virus in Malformed Newborn Calves
Source: Emerg Infect Dis. 2014 Aug;20(8):1327–30. doi: 10.3201/eid2008.121890 (PMC4111192; doi:10.3201/eid2008.121890)
Supplement: Technical Appendix 6 — Mechanistic hypotheses underlying central nervous system and muscle changes. [file 12-1890-Techapp-s6.pdf]

Article DOI: http://dx.doi.org/10.3201/eid2008.121890

Natural Intrauterine Infection with Schmallenberg Virus in Malformed Newborn Calves

Technical Appendix 6

Mechanistic Hypotheses Underlying Central Nervous System and Muscle Changes

Central Nervous System

From a mechanistic point of view, por- and hydranencephaly could result from destruction of the paraventricular germinal zone of the telencephalon, as well as of the neocortex that is already formed. The mechanisms involved can broadly be separated into 2 types that are not mutually exclusive: 1) direct cytopathogenicity of SBV on neuronal and glial cells and 2) ischemic necrosis. In the present Schmallenberg virus (SBV)–positive cases, there was very little evidence for ischemic destruction. In particular, neither thrombosis or perivascular edema, nor sequelae of hemorrhages (erythrophagocytosis or hemosiderin-laden macrophages) were seen, which sharply contrasts with histologic reports describing the similarly cavitated brains of bluetongue virus–infected bovine fetuses (1). Conversely, our histologic findings resemble those characterizing the cavitated telencephalon of lambs infected by Cache Valley virus in utero (2). Selective nerve cell necrosis and subsequent cavitation were, therefore, supposed to result from the direct cytolytic action of the virus itself. Necrosis of paraventricular immature, rapidly dividing neurons would prevent the outward migration of neuroblasts and subsequent maturation of the cerebral cortex and cause hypoplasia, whereas necrosis of established neocortical neurons would result in atrophy. In both cases, cavitation of necrotic areas then follows, the distinction between hydranencephaly and porencephaly being a matter of degree. A third subset of calves showed symmetrical dilatation of the lateral ventricles with corresponding thinning of the cortex (hydrocephaly). Logically, one of the causes of such damage is a stenosis of the mesencephalic aqueduct (3,4). When this happens, the lateral ventricles expand primarily at the expense of the neopallium (necrosis by compression) because the resistance of the diencephalon to mechanical constraints seems higher. The stenosis may result either from a developmental

disorder associated with a genetic defect (5) or from a prenatal or postnatal periependymal inflammation. It is tempting to invoke the latter mechanism here because a post-inflammatory stenosis of the aqueduct with secondary hydrocephaly has already been demonstrated in kittens and puppies infected in utero by the feline parvovirus (3) or canine parainfluenza virus, respectively (4). Unfortunately, we have not been able to confirm the existence of significant tissue remodeling around the aqueduct, which leaves the question of the origin of hydrocephaly unresolved. The systematic lack of signs of inflammation in the telencephalons yet highly deformed and in which the genetic material of the virus is still present is a remarkable feature of the cohort examined here. This absence suggests that infection occurred early during gestation, when the host response to infection is still limited and subsides to inapparent microscopic changes at term (6,7). Regarding the cerebellum, the observations gathered here (hypoplasia in a single calf) contrast with those reported recently by which about 40% of calves infected in utero displayed a cerebellar hypoplasia at necropsy (8). This difference remains unexplained.

The degree of overall body deformity was correlated with a progressively greater reduction in the size of the spinal cord (as determined by spinal cord:foramen magnum ratio) and with reduced numbers of spinal neurons, suggesting that the lack of movement leading to arthrogryposis is a direct consequence of the spinal cord lesions leading to denervation atrophy of skeletal muscle. This primary role for the spinal cord lesion is further supported by the tendency of forelimbs and hind limbs to be affected bilaterally because muscle involvement might be expected to lead to more randomly distributed lesions.

Skeletal Muscles

Taken together, the macroscopic and microscopic changes in skeletal muscles are compatible with several distinct lesions: 1) atrophy, 2) compensatory hypertrophy, 3) chronic polymyositis, 4) lipomatosis, and 5) attempts to regenerate. The coexistence of these lesions is characteristic of so-called end-stage muscles, i.e., muscles in which processes are long at work. This picture is consistent with denervation atrophy as aforediscussed, but a virus-induced necrotizing polymyositis could have contributed as well. In this respect, the detection of SBV genetic material in all skeletal muscles of animal E suggests that the fetal muscles are permissive to the virus that could cause necrosis by a direct cytolytic effect on myofibers as Akabane and Cache Valley viruses do (3,9). In addition, the question of why the virus was detected in the skeletal muscles of a single SBV-infected calf and never in the muscles of the other 14 remains unresolved.

References

1. MacLachlan NJ, Osburn BI. Bluetongue virus–induced hydranencephaly in cattle. Vet Pathol. 1983;20:563–73. PubMed http://dx.doi.org/10.1177/030098588302000508

2. Rodrigues Hoffmann A, Welsh CJ, Wilcox Varner P, de la Concha-Bermejillo A, Marchand Ball J, Ambrus A, et al. Identification of the target cells and sequence of infection during experimental infection of ovine fetuses with Cache Valley virus. J Virol. 2012;86:4793–800. PubMed http://dx.doi.org/10.1128/JVI.06858-11

3. Csiza CK, Scott FW, de Lahunta A. Feline viruses: XIV, transplacental infections in spontaneous panleukopenia of cats. Cornell Vet. 1971;61:423–39. PubMed

4. Baumgärtner WK, Krakowka S, Koestner A, Evermann J. Acute encephalitis and hydrocephalus in dogs caused by canine parainfluenza virus. Vet Pathol. 1982;19:79–92. PubMed http://dx.doi.org/10.1177/030098588201900111

5. Cho DY, Leipold HW. Congenital defects in the bovine central nervous system. Vet Bull. 1977;47:489–504.

6. Barlow RM. Morphogenesis of hydranencephaly and other intracranial malformations in progeny of pregnant ewes infected with pestiviruses. J Comp Pathol. 1980;90:87–98. PubMed http://dx.doi.org/10.1016/0021-9975(80)90031-6

7. Huxtable C, Jubb KVF. The nervous system. In Jubb KVF, Kennedy PC, Palmer NC, editors. Pathology of domestic animals. Vol 1. 4th ed. San Diego (CA): Academic Press; 1993. pp 198, 270–86.

8. Herder V, Wohlsein P, Peters M, Hansmann F, Baumgärtner W. Salient lesions in domestic ruminants infected with the emerging so-called Schmallenberg virus in Germany. Vet Pathol. 2012;49:588–91. PubMed http://dx.doi.org/10.1177/0300985812447831

9. Konno S, Nakagawa M. Akabane disease in cattle: congenital abnormalities caused by viral infection. Experimental disease. Vet Pathol. 1982;19:267–79. PubMed http://dx.doi.org/10.1177/030098588201900305
